# A Web-Based Geolocated Directory of Crisis Pregnancy Centers (CPCs) in the United States: Description of CPC Map Methods and Design Features and Analysis of Baseline Data

**DOI:** 10.2196/16726

**Published:** 2020-03-27

**Authors:** Andrea Swartzendruber, Danielle N Lambert

**Affiliations:** 1 Epidemiology and Biostatistics Department College of Public Health University of Georgia Athens, GA United States

**Keywords:** directory, crisis pregnancy center, abortion, induced, reproductive health, policy, access to information

## Abstract

**Background:**

Crisis pregnancy centers (CPCs) are nonprofit organizations that aim to dissuade people considering abortion. The centers frequently advertise in misleading ways and provide inaccurate health information. CPCs in the United States are becoming more medicalized and gaining government funding and support. We created a CPC Map, a Web-based geolocated database of all CPCs currently operating in the United States, to help individuals seeking health services know which centers are CPCs and to facilitate academic research.

**Objective:**

This study aimed to describe the methods used to develop and maintain the CPC Map and baseline findings regarding the number and distribution of CPCs in the United States. We also examined associations between direct state funding and the number of CPCs and relationships between the number of CPCs and state legislation proposed in 2018-2019 to ban all or most abortions.

**Methods:**

In 2018, we used standard protocols to identify and verify the locations of and services offered by CPCs operating in the United States. The CPC Map was designed to be a publicly accessible, user-friendly searchable database that can be easily updated. We examined the number of CPCs and, using existing data, the ratios of women of reproductive age to CPCs and CPCs to abortion facilities nationally and by region, subregion, and state. We used unadjusted and adjusted negative binomial regression models to examine associations between direct state funding and the number of CPCs. We used unadjusted and adjusted logistic regression models to examine associations between the number of CPCs by state and legislation introduced in 2018-2019 to ban all or most abortions. Adjusted models controlled for the numbers of women of reproductive age and abortion facilities per state.

**Results:**

We identified 2527 operating CPCs. Of these, 66.17% (1672/2527) offered limited medical services. Nationally, the ratio of women of reproductive age to CPCs was 29,304:1. The number of CPCs per abortion facility was 3.2. The South and Midwest had the greatest numbers of CPCs. The number of CPCs per state ranged from three (Rhode Island) to 203 (Texas). Direct funding was associated with a greater number of CPCs in unadjusted (coefficient: 0.87, 95% CI 0.51-1.22) and adjusted (coefficient: 0.45, 95% CI 0.33-0.57) analyses. The number of CPCs was associated with the state legislation introduced in 2018-2019 to ban all or most abortions in unadjusted (odds ratio [OR] 1.04, 95% CI 1.01-1.06) and adjusted analyses (OR 1.11, 95% CI 1.04-1.19).

**Conclusions:**

CPCs are located in every state and particularly prevalent in the South and Midwest. Distribution of CPCs in the United States is associated with state funding and extreme proposals to restrict abortion. Researchers should track CPCs over time and examine factors that influence their operations and impact on public health and policy.

## Introduction

### Background

Crisis pregnancy centers (CPCs, also known as *pregnancy resource centers* and *fake women’s health clinics*) are nonprofit organizations that primarily aim to dissuade people from seeking abortions [1,2]. Other aims include Christian evangelism and promoting sexual abstinence before marriage and marriage [2,3]. Most CPCs in the United States are affiliated with national organizations, such as Care Net and Heartbeat International, that have policies against promoting contraception [4]. CPCs have been operating in the United States since the 1960s and have traditionally provided pregnancy testing and counseling to influence individuals’ pregnancy decisions and discourage people from seeking abortion [5]. CPCs in the United States are increasingly becoming medicalized, offering limited medical services, such as limited obstetric ultrasounds to confirm pregnancy and testing for some sexually transmitted infections [6]. However, CPC services do not align with national quality family planning service recommendations that define a core set of services to prevent missed opportunities for comprehensive prevention and treatment [7]. CPCs also often fail to adhere to standard ethical principles [5], such as respect and responsibility. For example, to attract individuals who may not otherwise seek their services, CPCs frequently advertise themselves in misleading ways [5-8]. For example, the centers often give the appearance that they offer services that they do not provide, such as abortion [5-8]. CPCs also frequently provide biased, misleading, and inaccurate health information in support of their objectives [1,4,6-11]. In particular, CPCs frequently provide misleading and inaccurate information about the risks of abortion and misinformation about contraceptives and condom effectiveness [1,4,6-11].

CPCs in the United States have increasingly gained government funding and political clout [6,12]. CPCs have received federal grants to support abstinence-only education in public schools for decades [13,14]. An increasing number of states support CPCs through the sale of *Choose Life* license plates and directly fund the centers through dedicated grant programs [6,14]. The Trump Administration appointed multiple CPC proponents to leadership positions. For example, the current Deputy Assistant Secretary for Population Affairs (DASPA) within the Department of Health and Human Services was formerly President and Chief Executive Officer of a network of CPCs [12]. In 2018, the DASPA was provided final decision-making authority over which organizations receive Title X grants intended to provide family planning and related preventive services to low-income or uninsured individuals [15]. In 2019, the Trump Administration announced changes to the Title X program that made CPCs eligible for the federal grants, despite the fact that CPCs do not provide contraception, and awarded funding to a California-based CPC network [16]. CPCs were also awarded federal grant funding through the Teen Pregnancy Prevention Program in 2019.

In addition to government support and funding, CPCs in the United States have also won important legal protections. CPCs are not subject to the same regulatory requirements as health facilities and are largely unregulated [5,14]. California was the first state to pass state-level legislation aimed at regulating CPCs. The 2015 California Reproduction Freedom, Accountability, Comprehensive Care, and Transparency Act mandated that unlicensed CPCs disclose that the centers are not health facilities and licensed CPCs provide information about state programs that provide abortion, prenatal, and family planning services at little or no cost to eligible individuals. In 2018, although, in a 5-4 decision in *the National Institute of Family and Life Advocates (NIFLA) versus Bacerra*, the US Supreme Court ruled in favor of CPCs’ First Amendment rights and struck down the law [12].

To date, reported estimates of the total number of CPCs in the United States have widely varied. Antichoice groups’ estimate of 2500-4000 CPCs [6] has commonly been cited in scientific articles published since the early 2000s. A 2017 study that compiled publicly accessible directories maintained by national umbrella organizations such as Care Net, Birthright International, and NIFLA reported >4500 CPCs nationally [17]. However, the investigators did not assess data quality or verify information reported by the organizations. Other maps and directories of CPCs have also suffered from key limitations. For example, state-level directories, by definition, are limited in scope. Furthermore, methods for producing these directories are not readily accessible leading to questions about rigor and comparability. As previously mentioned, umbrella organizations that support CPCs maintain directories of affiliated centers, but none is comprehensive of all CPCs currently operating in the country. Other national maps and directories of CPCs have been produced but are limited because they are known to be incomplete, their methods have not been reported, it is unclear if the data have been verified, they are not searchable, or they are difficult to navigate. Despite increasing medicalization of CPCs, to date, no comprehensive database has categorized or estimated the number of CPCs that provide information only or limited medical services in addition to information.

Given that CPCs often employ misleading and deceptive advertising tactics, some people may visit CPCs with misconceptions about the centers’ mission and services [5]. Evidence suggests that CPC services may pose risk to individual and public health by impacting decision making about health behaviors and health care seeking and through delayed care [18]; however, evidence about CPCs’ impact is limited. Furthermore, CPCs’ role in the landscape of sexual and reproductive health services and abortion policy is not well understood. The number of facilities that provide abortion has declined over the past decade [19]. To date, no studies have compared the number of CPCs and facilities that provide abortion by state. Despite a rapidly changing policy environment, studies have not examined how government sponsorship influences the proliferation of CPCs or how CPCs might influence abortion policies. In 2018 and the first half of 2019, a record number of states introduced extreme legislation to ban all or most abortions [20-22]. As an active, grassroots part of the *pro-life* movement, a greater number of CPCs may signal a galvanized base of support for and potential legislative success in limiting abortion access.

### Objectives

We created a CPC Map, a Web-based geolocated database of all CPCs currently operating in the United States, with the following goals: (1) helping individuals seeking health services know which centers are CPCs and (2) facilitating academic research related to CPCs. Here, we describe the methods used to create and maintain the database, key design features of the tool and related operating procedures, and baseline findings regarding the number and distribution of CPCs in the United States. Specifically, we examined the number of CPCs nationally and by state, subregion, and region and in relation to the number of women of reproductive age and abortion facilities. We also investigated associations between direct state funding for CPCs and the number of CPCs per state and relationships between the number of CPCs and legislation proposed in 2018 and from January through July 2019 to ban all or most abortions.

## Methods

### Data Sources

Potential CPCs were identified through multiple internet searches conducted in March-May 2018, by trained research assistants following a standard protocol. All searches were conducted using Google search engine in incognito mode. First, we accessed five Web-based directories of CPCs to create an unduplicated list of CPCs by state: Care Net, Heartbeat International, NIFLA, Birthright International, and Ramah International [23-27]. For each entry, we recorded the center’s name, address, county, telephone number, and proprietary client-facing (ie, targeted to potential clients) website. If no website was provided, we searched for the site using the following keywords: [name of center], [city], and [state]. Next, we conducted keyword searches by separately entering [state] with “pregnancy resource center,” “crisis pregnancy center,” “pregnancy care center,” and “pregnancy center.” We reviewed the first five pages of results for each search (approximately 50 links per keyword search) and added unique entries to the master list. Next, we identified and reviewed existing maps by state to identify additional unique entries that were then added to the master list. We entered [state], “crisis pregnancy centers,” and “map” and reviewed the first two pages of entries (approximately 20 links). We also reviewed an existing crowd-sourced Web-based directory of CPCs by state and added unique entries to the master list [28]. Finally, we searched websites of listed entries for additional potential CPC addresses and added unique entries to the master list. Each search and entry were independently verified. For all entries, we recorded the method(s) by which the center was identified.

### Eligibility

From May to August 2018, trained research assistants evaluated each entry for eligibility and confirmed the name of the center and the center’s address. Centers were eligible for inclusion if they were determined to be (1) currently in business and (2) a CPC. Mobile clinics and maternity homes were excluded.

First, we examined if the recorded name of the center was the exact same as the name listed on the center’s website. If the center’s name was not exactly as it appeared on its website, we corrected the center’s name on the master list to match the name that appeared on the website. For centers with websites that did not clearly list the centers’ names and for which no proprietary website was identified, we called the centers to confirm their names using a standard script and protocol.

A center was categorized as currently in business if (1) its address was listed on a live propriety domain or (2) a respondent confirmed the center’s address during a telephone call to the center. Using a standard script and protocol, trained research assistants called all centers with addresses not listed on a proprietary domain. Centers with disconnected or out of service telephone numbers and those that could not be reached within five call attempts were categorized as not currently in business.

A center was categorized as a CPC if it (1) was identified through one of the search strategies, (2) advertised free pregnancy tests or testing and counseling on a live proprietary domain site or the center confirmed the availability of free pregnancy tests or testing during a telephone call to the center, (3) did not perform abortions or have *obstetrics/gynecology* in the site name, and (4) was not a family planning clinic or an informational directory that included local CPCs. Using a standard script and protocol, trained research assistants called all centers with websites that did not explicitly advertise free pregnancy tests or testing and centers with no identified client-facing proprietary website. Callers did not identify themselves as research assistants or explain the nature of the call.

### Types of Services

We also identified whether each eligible CPC provided information or counseling only or limited medical services in addition to information or counseling. CPCs that advertised free limited ultrasound services (excluding referrals) on a proprietary domain or confirmed the availability of free limited ultrasound services for any type or group of clients during a telephone call to the center were categorized as providing limited medical services. All other CPCs were categorized as providing information only.

### Design Features and Operating Protocols

The CPC Map’s design features reflect our goals to aid people in determining which centers are CPCs and facilitate research. Intended users included individuals seeking health services, public health and medical professionals, social service organizations, researchers, and decision makers. Key features include (1) accessibility and an open-source widget that allows distribution of the CPC Map on existing websites and apps, (2) faceted search, (3) geo-tracking to facilitate localized search results, (4) Google map and data visualization, (5) categorization of CPCs that provide information only vs limited medical services, (6) enumeration of CPCs, (7) marker clustering, (8) a webform to provide updates about included CPCs, (9) a webform to suggest a CPC not already included, and (10) a webform to request access to the CPC Map data set. Below, we describe these features and related protocols in greater detail.

The CPC Map is a national directory of CPCs that is publicly available [29]. The website, which is both desktop and mobile responsive, was publicly released on September 10, 2018. In addition, an open-source iFrame available on the site allows distribution of and access to the directory through existing websites and mobile apps. The directory, whether accessed through the main CPC Map website or widget display, is searchable by state, city, and zip code. Users who search by city or zip code are able to select radii of 5, 10, 25, 50, and 200 miles. CPC results can be presented in both map and list views. The homepage displays the map view with markers indicating locations of CPCs and includes a scroll panel that lists CPC names and addresses. Given the broadly recognized desire for and value of localized search results, the site includes geo-tracking, which, if allowed by the user, presents CPCs on the homepage at a resolution below city but above streets based on the user’s internet protocol address. A separate, searchable list view can be accessed via an icon on the homepage. Both the list and map views allow users to select presentation of CPCs that offer information only or limited medical services in addition to information, or all CPCs. CPCs that offer information only are indicated via blue markers, and centers that offer limited medical services in addition to information are indicated via green markers. All search results include the total number of centers in the geographic area selected. To aid visual representation of a large number of markers on the homepage map, which presents all CPCs currently operating in the United States, the CPC Map utilizes marker clustering, a grid-based clustering technique that groups CPCs within close proximity and displays the number of CPCs within each cluster. As the user zooms out, the groups consolidate. As the user zooms in, individual centers are marked.

We intend to review and update the site annually. The CPC Map website also includes several webforms to facilitate maintenance and accuracy of the directory over time. Through webforms, users may suggest centers that should be included in the directory and submit changes to information (eg, name and address changes and types of services offered) about listed centers. Information provided via the webforms is sent to an email address maintained by the research team. Upon receipt of information about additional centers that should be included, the research team verifies the suggested information and determines whether the center is eligible for inclusion using the process described above. Centers that meet existing eligibility criteria are then added to the directory by research team members who have rights-based permission to make changes. Similarly, upon receipt of suggested information changes for centers already included in the directory, research team members verify the submitted information and update the directory, as necessary.

One of the goals underlying development of the CPC Map is to facilitate high-quality academic research related to CPCs. Users can request access to the database via a webform available on the CPC Map website. Individuals requesting access to the database are asked to provide their first and last name, organization, reason requesting access as specifically as possible, email address, and phone number. Requests are considered on a case-by-case basis. Access to the database is intended to be used for research and program planning purposes only. For example, researchers may use CPC Map data as a sampling frame or use CPC Map data in analyses. Program planners may use the data to geographically target or inform their efforts.

### Usability Testing

Before finalizing the website, five individuals including sexual and reproductive health researchers, a sexual and reproductive health policy expert, an organizer at a nonprofit women’s organization, a public health student, and sexual and reproductive health care consumers conducted user testing. Testers were asked to attempt to complete six user tasks and report back on their experiences and any problems in completing the tasks. Feedback from the testers confirmed that the website and its functions were user-friendly and potential users were enthusiastic about the usefulness of the directory. Feedback was also used to finalize the site. For example, based on testers’ feedback, we added a link to the webform to suggest a center to the *Contact Us* page and added tooltips that hover above the map and list view icons to explain their functions.

### Data Analysis

We conducted analyses to describe the number of centers identified during data collection and final enumeration of eligible CPCs and distribution of CPCs in the United States. We also conducted analyses to examine policy factors related to CPCs, website user data, and search engine visibility. First, we used summary statistics to enumerate centers identified during collection and the number of CPCs currently operating in the United States, in total and by types of services offered. We also used descriptive statistics to assess the distribution of CPCs by region, subregion, and state. Next, we calculated the ratio of women of reproductive age (ages 15-49 years) to CPCs and the ratio of CPCs to abortion facilities nationally and by region, subregion, and state. Estimates of mid-year 2017 populations were obtained from the US Census Bureau [30]. The number of abortion facilities was obtained from a 2018 study that conducted a systematic Web-based search of abortion facilities in the United States [19].

Next, we examined policy factors related to the number of CPCs in each state and the District of Columbia. We examined the association between direct state funding for CPCs (yes/no) and the number of CPCs, a count variable, using unadjusted and adjusted mixed effect negative binomial regression models with a random intercept for region and robust standard errors. We used negative binomial regression models because analyses showed that Poisson models were not a good fit. Adjusted models controlled for the number of women of reproductive age and number of abortion facilities per state. Information about states that directly fund CPCs was obtained from a 2019 report released by a national advocacy organization [31]. States that directly funded CPCs (Florida, Georgia, Indiana, Kansas, Louisiana, Michigan, Minnesota, Missouri, New Mexico, North Carolina, North Dakota, Ohio, Oklahoma, Pennsylvania, Texas, and Wisconsin) were coded 1, and all others were coded 0.

We used unadjusted and adjusted logistic regression models to examine associations between the number of CPCs and state legislation to ban all or most abortions introduced in 2018 and from January through July 2019. Adjusted models controlled for the number of women of reproductive age and number of abortion facilities per state. We separately assessed associations between the number of CPCs and legislation to ban all or most abortions introduced in 2018, 2019, and in either year (2018-2019). Information about states that introduced legislation to ban all or most abortions was obtained from the Guttmacher Institute [20]. The following states introduced legislation to ban all or most abortions in 2018: Colorado, Illinois, Iowa, Indiana, Kentucky, Minnesota, Mississippi, Missouri, New Hampshire, New York, Ohio, Oklahoma, Pennsylvania, South Carolina, and Tennessee. States that introduced legislation to ban all or most abortions in 2019 included: Alabama, Arkansas, Georgia, Florida, Illinois, Indiana, Iowa, Kentucky, Louisiana, Maryland, Michigan, Minnesota, Mississippi, Missouri, New York, Ohio, Oklahoma, South Carolina, Tennessee, Texas, Washington, and West Virginia. States that introduced legislation were coded as 1; all others were coded as 0.

Finally, we used Google Analytics to describe the total number of views and unique views of the CPC Map within the first 10 months following release of the website. We also examined the number of domains that contained links to the CPC Map and the number that embedded the CPC Map widget. In addition, we used SEMRush to analyze search engine results and catalog relevant queries (keywords) with notable volume that drove organic traffic to the site. We then identified and quantified the number of queries that ranked on Google’s first page.

## Results

### Enumerating Crisis Pregnancy Centers

Using the multiple data sources described above, 4379 CPCs were initially identified through the search procedures. The compiled list was then reviewed for duplicate entries. A total of 14.20% (622/4379) of duplicate listings were identified, resulting in 3754 unique entries. These entries were then further reviewed for eligibility to determine if they were currently in business, offered free pregnancy tests or testing, and were a CPC. Of the unique sites found through the search procedures, 67.3% (2527/3754) were identified as eligible and operating CPCs. Of these, 66.17% (1672/2527) offered limited medical services in addition to pregnancy testing and counseling. Nationally, the ratio of women of reproductive age to CPCs was 29,304:1 per center. The number of CPCs per abortion facility was 3.2 nationally.

### Distribution of Crisis Pregnancy Centers in the United States

The distribution of CPCs varied across region (Table 1). The South had the greatest number of CPCs and the highest proportion of centers that offered limited medical services. The Northeast had the fewest CPCs and lowest proportion that offered limited medical services. The Midwest had the lowest ratio of women of reproductive age to centers, and the West had the highest. The Midwest had the highest ratio of CPCs to abortion facilities, and the Northeast had the lowest.

The distribution of CPCs also varied by state: Rhode Island, Delaware, and Hawaii were among the states with the fewest CPCs along with the District of Columbia. None of these was categorized as directly funding CPCs. The five states with the greatest number of CPCs included Texas, Florida, California, Pennsylvania, and Ohio. Of these, only California was categorized as not directly funding CPCs.

States with the highest proportion of centers that provided limited medical services included Rhode Island, Louisiana, Nevada, North Dakota, and Delaware. States with the lowest proportion included District of Columbia, Connecticut, New York, Vermont, and Maine. Wyoming, Montana, Iowa, South Dakota, and Kansas had the lowest ratio of women of reproductive age to CPCs, whereas New Mexico, District of Columbia, Nevada, Rhode Island, and California had the highest.

In only two states, Massachusetts and New Jersey, and the District of Columbia, the ratio of CPCs to abortion facilities was less than 1. There were approximately equal numbers of CPCs and abortion facilities in California and Rhode Island. In all other states, CPCs outnumbered abortion facilities. The ratio was highest in Missouri, Kentucky, and Mississippi, each of which had only a single abortion facility.

**Table 1:** Number of crisis pregnancy centers in the United States, by region and state, in 2018.

Region and state			CPCs^a^, n		CPCs that offer limited medical services, n (%)		Population of women of reproductive age (ages 15-49 years) per CPC, n		Ratio of CPCs to abortion facilities	
United States			2527		1672 (66.17)		29,304		3.2	
**Northeast**			352		168 (47.7)		36,820		1.5	
	**New England**		83		40 (48)		40,706		1.1	
		Connecticut		21		7 (33)		38,613		1.1
		Maine		11		5 (46)		25,445		0.6
		Massachusetts		25		11 (44)		64,611		1.3
		New Hampshire		15		11 (73)		19,360		2.5
		Rhode Island		3		3 (100)		82,094		1.0
		Vermont		8		3 (37)		16,985		1.3
	**Middle Atlantic**		269		128 (47.6)		35,621		1.7	
		New Jersey		37		22 (59)		55,330		0.7
		New York		107		37 (34.6)		44,028		1.2
		Pennsylvania		125		69 (55.2)		22,591		7.4
**Midwest**			724		474 (65.5)		21,073		7.9	
	**East North Central**		455		321 (70.6)		23,234		6.7	
		Indiana		96		73 (76)		15,688		16.0
		Illinois		86		59 (68)		34,859		3.4
		Michigan		99		64 (64)		22,339		4.3
		Ohio		119		85 (71.4)		21,724		10.8
		Wisconsin		55		40 (72)		23,110		18.3
	**West North Central**		269		153 (56.9)		17,417		11.2	
		Iowa		49		28 (57)		13,911		5.4
		Kansas		36		17 (47)		17,880		9.0
		Minnesota		77		39 (51)		15,969		15.4
		Missouri		69		47 (68)		19,769		69.0
		Nebraska		20		12 (60)		21,037		6.7
		North Dakota		7		6 (86)		23,446		7.0
		South Dakota		11		4 (36)		16,484		11.0
**South**			1003		745 (74.28)		28,031		5.2	
	**South Atlantic**		486		361 (74.3)		29,906		3.3	
		Delaware		6		5 (83)		35,298		2.0
		District of Columbia		2		0 (0)		99,643		0.7
		Florida		160		132 (82.5)		27,590		2.7
		Georgia		90		70 (78)		27,540		5.3
		Maryland		48		38 (79)		29,464		1.9
		North Carolina		83		60 (72)		28,253		5.5
		South Carolina		32		18 (56)		34,763		10.7
		Virginia		51		31 (60)		38,600		3.4
		West Virginia		14		7 (50)		27,850		14.0
	**East South Central**		200		142 (71.0)		21,642		13.3	
		Alabama		52		39 (75)		21,452		10.4
		Kentucky		54		34 (63)		18,477		54.0
		Mississippi		29		18 (62)		23,929		29.0
		Tennessee		65		51 (78)		23,403		8.1
	**West South Central**		317		242 (76.3)		29,188		10.2	
		Arkansas		37		27 (73)		18,095		12.3
		Louisiana		29		25 (86)		37,452		9.7
		Oklahoma		48		37 (77)		18,324		12.0
		Texas		203		153 (75.4)		32,597		9.7
**West**			448		285 (63.6)		39,656		1.7	
	**Mountain**		196		128 (65.3)		27,370		3.6	
		Arizona		53		35 (66)		28,786		6.6
		Colorado		58		38 (66)		22,009		2.8
		Idaho		19		11 (58)		19,270		4.8
		Montana		18		11 (61)		12,017		3.6
		Nevada		7		6 (86)		95,375		0.9
		New Mexico		22		13 (59)		20,849		4.4
		Utah		7		4 (57)		104,029		3.5
		Wyoming		12		10 (83)		10,441		6.0
	**Pacific**		252		157 (62.3)		49,212		1.2	
		Alaska		9		5 (56)		18,790		1.5
		California		147		93 (63.3)		63,665		1.0
		Hawaii		6		4 (67)		51,811		2.0
		Oregon		43		26 (60)		21,214		3.6
		Washington		47		29 (62)		35,117		1.4

^a^CPC: crisis pregnancy center.

### Policy Analyses

We found significant positive associations between direct state-level funding for CPCs and the number of centers in states in both unadjusted (coefficient: 0.87, 95% CI 0.51-1.22; *P*<.001) and adjusted models (coefficient: 0.45, 95% CI 0.33-0.57; *P*<.001). Table 2 presents associations between the number of CPCs in each state and the District of Columbia and legislation to ban all or most abortions proposed in 2018 and through July 2019. A greater number of CPCs was positively associated with legislation to ban all or most abortions introduced in 2018, 2019, and 2018-2019 in both unadjusted and adjusted analyses.

**Table 2:** Associations between the number of crisis pregnancy centers in each state and the District of Columbia and legislation proposed in 2018 and January-July 2019 to ban all or most abortions.

The year in which legislation to ban all or most abortions was introduced	Unadjusted analysis			Adjusted^a^ analysis	
	OR^b^ (95% CI)	*P* value	OR (95% CI)		*P* value
2018	1.01 (1.00-1.03)	.09	1.08 (1.02-1.14)		.005
2019	1.03 (1.01-1.05)	.004	1.06 (1.01-1.12)		.01
2018 or 2019	1.04 (1.01-1.06)	.002	1.11 (1.04-1.19)		.002

^a^Adjusted for the number of abortion facilities and women aged 15 to 49 years per state.

^b^OR: odds ratio.

### Website Analytics

With no paid advertising, the CPC Map website received 9516 unique views and 11,872 total views in the initial 10 months after release, and views steadily increased over time. During the same period, 177 domains contained links to the CPC Map, including major and regional news outlets. In July 2019, the CPC Map ranked for more than 3100 keywords, indicating a very high degree of relevant and valuable content. The CPC Map ranked for 13 terms with significant search volume on Google’s first search engine results page. For example, the site ranked sixth for *crisis pregnancy center near me* and *crisis pregnancy locations*, seventh for *what are CPCs*, and eighth for *teen pregnancy center near me*. Searches that include *near me* indicate strong signals of user intent and suggest that the CPC Map is successfully reaching people seeking to identify local CPCs.

## Discussion

### Principal Findings

Individuals facing or at risk for unintended pregnancy require quality sexual and reproductive health information and services. CPCs frequently provide inaccurate health information and do not adhere to medical or ethical practice standards, which could pose risk to individual and public health [18]. CPCs are becoming more medicalized and increasingly gaining government support. The purpose of the CPC Map is to identify the number and locations of CPCs currently operating in the United States. We identified over 2500 CPCs currently operating in the United States, about two-thirds of which offered limited medical services. However, the distribution of centers was not uniform by region or state.

The South and Midwest had the highest numbers of CPCs and lowest ratios of reproductive-aged women to CPCs. We found that state funding was positively associated with a greater number of CPCs per state. In total, 88% (14/16) of the states that directly fund CPCs were located in the South and Midwest. As this study is cross-sectional, temporality cannot be established. It is currently unknown whether state funding attracts more centers or whether states with more centers are more successful in attracting state funding. Over time, the CPC Map may be useful for longitudinally tracking how the number of CPCs changes and the potential impact of state government support. That approximately one-third of states directly fund the centers despite lack of evidence of public health benefit and potential risks point to additional factors that may also influence the numbers and locations of CPCs. Political climate and religious context likely underlie whether states directly fund CPCs, the number of CPCs, and the ratio of CPCs to abortion facilities in a state. Future studies that more fully explore state-level factors related to the number of CPCs per state and changes over time would be helpful to better understand contexts that limit and facilitate CPC operations.

Nationally, there are over three times as many CPCs as abortion facilities. In only four states and the District of Columbia, the ratio of CPCs to abortion facilities was approximately 1 or less, suggesting that in most of the United States, people have better access to CPCs than abortion care. Access to abortion is a function of residence. The Midwest and South have the fewest abortion facilities [19] and greatest number of CPCs resulting in nearly eight times as many CPCs in the Midwest and over five times as many in the South.

We also found that a greater number of CPCs was associated with state abortion bans introduced in 2018 and 2019. An unprecedented wave of legislation restricting access to abortion has been enacted since 2011 [21]. Following Supreme Court changes, 2019 marked a new level of proposed legislation to ban abortion [22]. The current findings show that a greater number of CPCs predicted the most extreme legislation introduced in 2019 that aimed to ban all or most abortions, including legislation to ban abortion completely and to ban abortion after 6 to 8 weeks of gestation. CPCs are one facet of a movement eager to make abortion unlawful nationally. Although this study was not able to thoroughly explore factors associated with where and what types of abortion bans were introduced, CPCs may represent a significant base of support and mobilization for this type of legislation. What impact such bans and other abortion restrictions, if enacted and implemented, would have on the number of CPCs in each state is unknown. If abortion was completely banned in only some states, CPCs may strategically focus their efforts in states where abortion remained legal. Alternatively, CPCs may see their objectives of promoting sexual abstinence before marriage and childbearing as unchanged or perhaps perceive an even greater need for their pregnancy support services if abortion became illegal in some states or nationally. The CPC Map is well suited to track these potential changes over time and to facilitate analyses related to how state policy environments are influenced by and influence CPCs.

### Strengths and Limitations

The CPC Map is subject to several limitations. Although our team followed standard protocols to create the tool, the CPC Map is dependent on the accuracy of publicly available information about centers and their locations. Rigorous data collection occurred in April-June 2018. Although we intend to maintain the CPC Map over time, the tool is not updated constantly, and we cannot guarantee the completeness and accuracy of the CPC Map, particularly as CPCs do change names and locations and increasingly offer limited medical services. However, the CPC Map’s design facilitates a process for obtaining and verifying updates submitted by users. In addition, the current analysis focused on between-state comparisons. Investigating locations of CPCs within states might also be important for better understanding factors that influence where CPCs operate, groups that might be most impacted by CPC services, and access to sexual and reproductive health services and information in different areas. For example, examining factors such as proximity to schools, racial composition of the population, rural and urban differences, and proximity to hospitals, abortion facilities, and other sources of health care may provide further insights about where CPCs locate, contexts that facilitate and constrain CPC operations, and individuals and groups that might be most impacted by CPC services. Finally, although our adjusted analyses controlled for multiple potential confounders, the findings may be limited by unidentified sources of confounding, which may have led to inflated or underestimated results.

### Conclusions

In an era of volatile policy dynamics and intense change related to sexual and reproductive health care access and rights, the CPC Map was designed to help raise awareness about CPCs and track the extent to which CPCs change in number, location, and types of services offered over time. The purpose of the CPC Map was to create an accessible, user-friendly Web-based geolocated database of all of the CPCs operating in the United States to help make sexual and reproductive health care consumers aware of which centers are CPCs and to facilitate and grow the evidence base related to CPCs, particularly in a period when CPCs are benefitting from significant US government support and funding. Direct, organic, and referral traffic to the site incrementally increased since the release of the CPC Map despite no paid advertising, indicating increasing reach and potentially increased awareness about CPCs and their locations. This study revealed that CPCs are located in every state and are particularly prevalent in the South and Midwest, which also have the fewest abortion facilities. Nationally, CPCs outnumber abortion facilities by a factor of 3.2. We found that state funding for CPCs was positively associated with the number of CPCs, and a greater number of CPCs predicted introduction of extreme state legislation restricting abortion. Given increasing government investment in CPCs, researchers should continue to track CPCs and examine factors that influence CPCs’ operations, strategies, and impact on public health and policy.

## Abbreviations

CPC: crisis pregnancy center
DASPA: Deputy Assistant Secretary for Population Affairs
NIFLA: National Institute of Family and Life Advocates
OR: odds ratio
